# A Role for Advanced Glycation End Products in Molecular Ageing

**DOI:** 10.3390/ijms24129881

**Published:** 2023-06-08

**Authors:** Katarzyna Zgutka, Marta Tkacz, Patrycja Tomasiak, Maciej Tarnowski

**Affiliations:** 1 Department of Physiology in Health Sciences, Faculty of Health Sciences, Pomeranian Medical University, Żołnierska 54, 70-210 Szczecin, Poland; 2 Institute of Physical Culture Sciences, University of Szczecin, 70-453 Szczecin, Poland

**Keywords:** AGE, ageing, oxidative stress, inflammation, brain, age-related diseases, caloric restriction, physical activities

## Abstract

Ageing is a composite process that involves numerous changes at the cellular, tissue, organ and whole-body levels. These changes result in decreased functioning of the organism and the development of certain conditions, which ultimately lead to an increased risk of death. Advanced glycation end products (AGEs) are a family of compounds with a diverse chemical nature. They are the products of non-enzymatic reactions between reducing sugars and proteins, lipids or nucleic acids and are synthesised in high amounts in both physiological and pathological conditions. Accumulation of these molecules increases the level of damage to tissue/organs structures (immune elements, connective tissue, brain, pancreatic beta cells, nephrons, and muscles), which consequently triggers the development of age-related diseases, such as diabetes mellitus, neurodegeneration, and cardiovascular and kidney disorders. Irrespective of the role of AGEs in the initiation or progression of chronic disorders, a reduction in their levels would certainly provide health benefits. In this review, we provide an overview of the role of AGEs in these areas. Moreover, we provide examples of lifestyle interventions, such as caloric restriction or physical activities, that may modulate AGE formation and accumulation and help to promote healthy ageing.

## 1. Introduction

Advanced glycation end products (AGEs) are heterogeneous molecules formed through non-enzymatic glycation of biological macromolecules, mostly in hyperglycaemic conditions. Non-enzymatic glycation occurs through a series of chemical reactions between the active carbonyl groups of reducing sugars and free amines of nucleic acids, proteins, and lipids. AGEs are potentially toxic molecules that threaten human health because they negatively impact several tissues through the formation of reactive oxygen species (ROS), generation of aberrant proteins or growth factors, alteration of the extracellular matrix (ECM) structure and secretion of pro-inflammatory cytokines [1,2]. The AGE concentration is controlled mostly by glucose levels; hence, it is difficult to differentiate the effects of hyperglycaemia from those of AGEs [3]. Additionally, various environmental factors, including cigarette smoke, high levels of refined and simple carbohydrates, hypercaloric diets, foods cooked at a high temperature, and a sedentary lifestyle, induce AGE production, and consequently affect various physiological processes [3,4].

Researchers have proposed a number of theories to explain the role of AGEs in various diseases and disorders. In one theory, AGEs bind to receptors of advanced glycation end products (RAGE)—the AGE–RAGE axis—and trigger a cascade of reactions and intracellular signalling pathways that affect proliferation, autophagy, apoptosis, and the inflammatory response [5,6,7]. In another theory, AGEs can cross-link ECM proteins and lipids, thus disturbing the structure and mechanical properties of tissues. Moreover, AGEs can promote oxidative stress, ROS generation and mitochondrial function impairment [8,9]. The damage caused by oxidative stress becomes exacerbated if the antioxidant enzymes themselves are inactivated by glycation [10]. Lipids, connective tissue ECM elements and nucleic acids are all potential substrates for AGE formation.

AGE accumulation leads to dysregulation of cellular physiology and cellular signalling pathways; it changes protein molecular structure, altering enzyme or receptor functioning and activity, and hence performs a vital role in the development of several diseases [11]. Moreover, AGEs promote oxidative stress and activate several stress-induced transcription factors, including the mitogen-activated protein kinase (MAPK), nuclear factor kappa B (NF-κB) and signal transducer and transcriptional activator (STAT) pathways [4]. The results are increased production of pro-inflammatory molecules, such as cytokines, chemokines, and acute-phase proteins. Endogenous anti-stress mediators are important for the management of high AGE levels, but they are not sufficient to control the progression of AGE-related pathologies [5]. It is crucial to modify the balance of AGE levels through exogenous mechanisms, such as proper nutrition, reducing smoking or adequate interventions [5,12]. Researchers have shown that AGEs perform a pathological role in the development and progression of some metabolic diseases, including diabetes mellitus (DM) [13,14,15,16], cardiovascular diseases (CVD) such as atherosclerosis [2,13,17,18,19], neurodegenerative diseases, such as Alzheimer’s disease (AD) [13,14,20,21,22] and particular types of cancer [23].

Ageing is characterised by a disturbance of anatomical integrity and function at the cellular, tissue and organ levels and a reduced ability to respond to stress. It is caused by the progressive accumulation of damage over time and decreased regenerative power. Ageing is a highly complex, multifactorial process where genetic, endogenous, and environmental factors exert their effects [24]. More than 300 theories of ageing have been proposed, including the progressive impairment of mitochondrial function, increased oxidative stress (the free radical theory) and immune activation [25,26]. It is very intriguing that these processes can be influenced and, to some extent, modified by adequate, optimised nutrition. Multiple studies on animal species have found that caloric restriction (CR) reduces oxidative stress and is associated with a longer life expectancy [27,28,29,30]. It has been shown that AGEs accumulate during normal ageing and with age-related diseases [3,31,32].

In this review, we focus on molecular changes and the involvement of multiple signalling pathways in AGE-related tissue degradation during ageing. It is naïve to consider AGEs to be the sole cause of ageing; however, researchers have postulated that the progressive in vivo rise in AGE levels—which occurs as part of normal ageing—increases the ability of AGEs to cross-link proteins in an irreversible fashion and their ability to trigger a plethora of cellular pathways to ‘accelerate’ ageing [33]. We summarise and update the knowledge on the harmful effects of AGEs on tissues and cells as demonstrated by molecular, in vitro, and animal studies. We evaluate the consequences of increasing exposure to exogenous AGE and possible interventions to decrease the harmful effects of AGEs.

## 2. AGEs

### 2.1. AGE Formation and Biochemistry

AGEs are formed via a multistep molecular process involving simple and complex chemical reactions. AGEs were first discovered in the early 1900s as the products of the non-enzymatic Maillard reaction, in which amino acids are heated in a mixture with reducing sugars, and the products of the reaction become yellowish-brown melanoids. We observe this process as the transformation of the colour of food that is cooked, grilled, or fried. In cuisine, it serves to enhance flavour and colour, giving a richness in taste and aroma to thermally processed foods with high sugar and protein content. AGEs were initially identified in cooked foods [34] and named by Brownlee et al. [35] in 1984.

**Figure 1:**
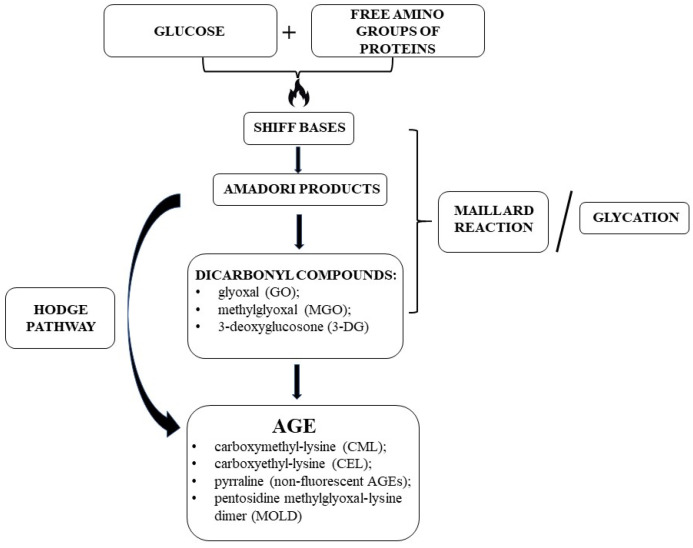
Formation of AGEs through nonenzymatic reactions of reducing sugars with free amino groups of proteins.

As mentioned above, the synthesis of AGEs involves multiple steps, where reducing sugars containing carbonyl groups—i.e., glucose—react non-enzymatically with free amino groups of proteins and nucleic acids to form unstable products, known as Schiff bases, an early glycation product (Figure 1). The driving force of this reaction depends on the glucose concentration. Next, Amadori products (stable ketoamine)—intermediate or early glycation products—are formed. Some Amadori products may be directly transformed into AGEs via irreversible oxidation or hydrolysis through a series of reactions known as the Hodge pathway [36]. Other Amadori products may be converted to AGE precursor compounds, such as glyoxal (GO), methylglyoxal (MGO) and 3-deoxyglucosone (3-DG) by dehydration, oxidative cracking, or cyclisation. Stable AGEs are formed by the covalent binding of active α-dicarbonyl compounds to long-lived proteins and structural components of the connective tissue matrix or basement membrane, such as collagen (Figure 1). These compounds may participate in oxidation, dehydration, or polymerisation reactions to give rise to numerous other AGEs [37,38].

More than 20 different types of AGEs have been identified in human blood and tissues and in foods [9,39]. In summary, AGEs can be divided into fluorescent and nonfluorescent groups. The most important ones include carboxymethyl-lysine (CML), carboxyethyl-lysine (CEL) and pyrraline (non-fluorescent AGEs), and pentosidine and methylglyoxal-lysine dimer (MOLD) (fluorescent AGEs) [9,39]. Fluorescent AGEs comprise a large proportion of AGEs and are a good target for measuring total AGEs [40]. Although they have diverse chemical structures, their common characteristic is the presence of a lysine residue. In initial investigations, the first observed endogenous product of glycation, glycated haemoglobin (HbA1c), was detected in 1968 in a person with DM [41]. The mechanisms of its formation include the addition of glucose molecules to amino groups located on haemoglobin β-chains with the formation of a Schiff base that is an unstable structure driving an Amadori rearrangement. The final product, 1-deoxy-1-fructosyl residue, has a carbohydrate fragment attached to HbA1c. In recent years, a number of techniques were developed to detect and measure AGEs’ concentration, including spectrofluorimeter, mass spectrometry (MALDITOF-MS), high-performance liquid chromatography (HPLC), SDS-PAGE analysis, and peptide mass fingerprinting methods or combinations of thereof. Moreover, typical methods of AGEs’ monitoring involve body fluids enzyme-linked immunosorbent assay (ELISA) and the measurement of autofluorescence of AGEs in skin as a non-invasive approach. What is important is that there is no universal method available, and the conversion from one to the other is troublesome and most commonly leads to inaccuracy [39,42].

AGEs can be formed through either exogenous or endogenous mechanisms. Exogenous AGEs come mainly from the diet; the highest levels of AGEs are found in animal-based foods with high fat and protein contents, such as canned meats; fried, grilled, and barbecued foods; and grain-based baked foods. Coffee, butter, vegetables, and fruits, and food prepared by steaming or boiling contain the lowest amounts of AGEs [43,44,45]. Of note, heat-treated foods are 10–100 times richer in AGEs than untreated foods [43]. The AGEs burden is the sum total of AGEs from dietary sources and endogenous synthesis. However, the relative contribution of endogenous versus exogenous AGEs in determining the total body AGE load and its physiological relevance is difficult to assess. The variability in AGE molecules; their differing bio-potential; and the lack of reliable data on their metabolism, absorption, and distribution in body compartments are the main research problems [15,16,17,18,19,20,21,22,23,24,25]. CML is a stable and relatively inert molecule that is used as a standard representative to assess AGE levels in food products [45]. It is estimated that the average amount of AGEs consumed on a daily basis by an individual range from 12,000 to 20,000 kilo-units (kU) with a median of 15,000 kU [4,45]. When the diet is rich in grilled or roasted meats, fats, and highly processed foods, the consumption could reach >20,000 kU AGEs/day [45]. Interestingly, protein-bound pentosidine is not as readily absorbed as free pentosidine; therefore, increased levels of free AGEs in urine and plasma are correlated to AGE-rich dietary intake and urinary pentosidine levels are clinically used to evaluate various AGE-related and ageing-associated disorders [46,47,48].

### 2.2. Endogenous and Exogenous AGEs: Balancing the AGE Content

Most endogenous AGEs are formed spontaneously by normal metabolic processes of the organism [49]; as mentioned above, they accumulate in the body during normal ageing. MGO is the most common endogenous mediator of AGE synthesis; this highly reactive dicarbonyl compound is formed as a by-product of glycolysis and is present ubiquitously in all cells. MGO is largely derived as a result of carbohydrate, lipid, or amino acid metabolism involving both enzymatic and non-enzymatic reactions [50,51,52]. MGO synthesis is catalysed by MGO synthase via the fragmentation of triosephosphates—glyceraldehyde-3-phosphate (GAP) and dihydroxyacetone phosphate (DHAP)—but also cytochrome P450 2E1, myeloperoxidase, and amino oxidase. These enzymes participate in glycolytic bypass, acetone metabolism, and amino acid breakdown, respectively. In hyperglycaemia, impaired glucose utilisation and triosephosphate isomerase deficiency led to MGO elevation [53]. MGO is one of the most potent glycating agents present in cells; thus, its accumulation is highly deleterious. MGO readily reacts with lipids, nucleic acids, and lysine and arginine residues of proteins to form AGEs, such as argpyrimidine and MGO-derived hydroimidazolone (MG-H1) [54].

What is physiologically relevant is how the body itself regulates the AGE content through detoxication and excretion via the kidneys, as the AGE levels are not only defined by the rate of their formation but also by the rate of their removal. In healthy people with normal kidney function, around 30% of ingested AGEs are excreted in the urine [55]. In individuals with diseases, such as DM or kidney dysfunction, <5% of AGEs are excreted in the urine [55,56]. Human and animal studies show that AGE elimination by the kidneys is limited and does not always correspond to AGEs ingestion. There is still no consensus regarding the gut absorption, tissue distribution, and elimination of AGEs via faeces and urine. There are contradictory results, and the methods for estimating AGE trafficking are underdeveloped. Nevertheless, it is widely accepted that AGE concentrations are commonly inversely related to renal function [57,58,59], and deterioration of kidney function leads to accumulation of AGEs and—what is critical here—this accumulation makes this organ vulnerable to AGE-mediated damage [57,58,59].

Environmental factors, such as excessive and/or prolonged alcohol consumption, cigarette smoking, the intake of high fat/caloric diets and/or extensively processed food, renal status, homeostatic imbalance, inflammation, hyperglycaemia, and oxidative stress influence the rate of AGE formation [60,61]. Specifically, a persistent state of hyperglycaemia, as seen in patients with type 2 diabetes mellitus (T2DM), enhances the process of glycation, accelerates AGE formation, and promotes additional biochemical abnormalities [4,33,60,61]. The story gets more complicated, given that healthy monozygotic and heterozygotic twins show that the levels of circulating AGEs seem to be genetically determined [62].

In addition to renal and faecal removal of AGEs, cells of the body are equipped with intrinsic detoxifying pathways against the accumulation of AGEs, such as the glyoxalase (Glo), aldose reductase, aldehyde dehydrogenase, and carbonyl reductase pathways. The glutathione-dependent Glo system, comprising Glo1 and Glo2, has a key role in the defence against glycation. This system uses reduced glutathione (GSH) to catalyse the conversion of GO, MGO, and other α-oxoaldehydes to the less toxic D-lactate [63]. Glo1 catalyses the metabolism of dicarbonyl compounds and prevents them from binding to proteins, protecting them from AGE formation [63,64]. Other enzymatic systems include fructosamine 3-kinase, fructosamine oxidase, 2-oxaldehyde reductase, and carbonyl reductase [65], which act to phosphorylate and destabilise Amadori products, leading to their spontaneous breakdown [66]. The levels of AGEs in the body are not only determined by the pace at which they are formed but also by their capacity to be eliminated by intrinsic detoxifying mechanisms. However, during ageing and under pathological conditions, these defence systems are unable to properly prevent AGE production.

It is very important to summarise that the imbalance in the AGE levels is a result of both endogenous synthesis and exogenous intake, and the amounts of ingested AGEs usually affect the concentration of AGEs in the body [60]. As described previously, AGE-rich foods and beverages are key triggers in AGE accumulation and, together with endogenous AGEs, synergistically stimulate oxidative stress, inflammation, and cellular damage. Increased availability of intracellular sugars, such as glucose-6-phosphate in the glycolytic pathway, speeds up glycation, and the formation of Amadori products. AGE accumulation and both hyperglycaemia and hyperlipidaemia cause a cumulative metabolic burden [67,68,69], ultimately promoting ongoing pathogenic stimulation.

## 3. AGEs: Mechanism of Action

### 3.1. The AGE–RAGE Interaction

AGEs act by modifying proteins or forming adducts with them (i.e., CML, pentosidine, or hydroimidazolone) through binding with AGE ligand-gated receptors, such as RAGE [39,70]. RAGE is a multiligand cell surface receptor belonging to the immunoglobulin superfamily; it is encoded by the gene AGER, located on chromosome 6 near the major histocompatibility complex III [71]. The gene consists of 11 exons, and typical variations in this gene have been described [72]. The receptor can also be found as a secreted soluble isoform (sRAGE) produced from truncated variants (described in Section 3 and Section 4). RAGE is a transmembrane protein that consists of an extracellular region characterised by type V1, C1, and C2 immunoglobulin domains; a transmembrane-spanning domain; and a short cytosolic tail [73,74]. The extracellular portion of RAGE is composed of a variable (V-type) domain, which is followed by two constant (C-type) domains and represents the main binding sites for various ligands, while the cytosolic tail of RAGE is essential for signalling [73]. The V1 and C1 domains of RAGE bind a large variety of molecules—not only AGEs (endogenous or food derived) but also advanced oxidation protein products (AOPPs) involved in oxidative stress [75], β-amyloid related to AD [73,74], calcium-binding S100 proteins linked to several human cancers [76] and high-mobility group box-1 (HMGB) expressed in cancer and inflammation [75,77].

RAGE has been identified in several organs and tissues, but the highest concentration is in the lung, heart, and skeletal muscles. Furthermore, RAGE is expressed on a wide range of cells, including smooth muscle cells, monocytes, macrophages, endothelial cells, astrocytes, and microglia [73]. Under physiological conditions, RAGE is expressed at basal levels; however, its levels are elevated in pathological conditions, such as DM, CVD, AD, cancer, and natural ageing [78,79].

The activation of RAGE induces an inflammatory cascade that starts with the activation of a transcription factor NF-κB that promotes the expression of pro-inflammatory cytokines, growth factors, and adhesion molecules [80,81]. Furthermore, RAGE activation by AGEs or other ligands transduces multiple signals, such as the MAPK, extracellular signal-regulated kinases 1 and 2 (ERK1/2), p21ras, p38, and Janus kinase (JAK)–STAT [82]. Activation of the various signalling pathways increases the transcription of many pro-inflammatory genes coding for interleukins (IL) and growth factors, including IL-1, IL-6, IL-8, monocyte chemoattractant protein-1 (MCP-1), and tumour necrosis factor alpha (TNF-α), and upregulation of adhesion molecules, such as vascular cell adhesion molecule (VCAM) and intracellular adhesion molecule (ICAM) [9,83]. These molecules promote inflammatory, proliferative, angiogenic, oxidative, fibrotic, thrombogenic (pro-coagulant), and apoptotic reactions [80,81,82,83,84,85].

### 3.2. AGEs Trigger Oxidative Stress

The AGE–RAGE interaction increases the levels of ROS through the activation of nicotinamide adenine dinucleotide phosphate oxidase (NADPH oxidase) and mitochondrial pathways [86]. ROS accumulate in tissues from the conversion of glucose to fructose through the NADPH pathway, leading to increased production of AGEs and a vicious cycle of intracellular damage. Consequently, the activity of superoxide dismutase (SOD), catalase, and other endogenous antioxidant defence mechanisms is decreased, such as GSH and ascorbic acid [87]. In fact, oxidative stress is highly related to glycation because the depletion of GSH also reduces the activity of Glo-1, thus increasing concentrations of GO and MGO [88]. Furthermore, AGEs increase the oxidation of low-density lipoprotein (LDL) and promote the development of atherosclerosis [89,90]. Therefore, glycated LDLs are more sensitive to oxidation [90]; they are reduced with difficulty and promote the formation of antibodies that bind AGEs located in vessel walls, which amplify the development of vascular inflammation and atherosclerosis [91]. Glo-1 overexpression has beneficial vascular effects, reduces ROS, and protects against atherogenic LDL formation [92]. In patients with T2DM, circulating AGE levels are positively correlated with RAGE messenger RNA (mRNA) expression and oxidative markers, such as protein carbonyl, AOPP generation, and lipid peroxidation [93].

### 3.3. Impact of AGEs on the ECM and Ageing

Human ageing is associated with important changes in the biomechanical properties of tissues, including stiffening and disturbed regeneration. These changes are especially relevant for tissues that are rich in ECM molecules and long-lived proteins, such as skeletal muscle, tendons, joints, bone, the heart, arteries, the lungs, skin, and the lens [94]. AGE accumulation is thought to depend on the protein turnover rate; therefore, long-lived proteins, such as collagen, are mainly modified by glycation. Moreover, sterile inflammation induced by increased AGE levels has a significant impact on the basement membrane, through the elevated levels of inflammatory cytokines, such as IL-1β and TNF-α [95]. They induce the production of ECM proteins and further stimulate local and general inflammatory responses. AGE covalent cross-linking to collagen or elastin in the ECM causes the collagen to be less susceptible to hydrolytic breakdown, and it becomes less flexible [96,97]; subsequently, the basement membrane begins to thicken. These changes affect the mechanical properties of tissues, especially of the vasculature [98,99], leading to increased rigidity of the aorta, the carotid arteries, and other larger arteries [100]. Regarding the cardiovascular system, the thickening of the basement membrane hinders the integrity of the blood vessels and increases the risk of haemorrhagic pathologies. Moreover, high AGE accumulation occurs in parallel to skin ageing. Skin collagens, mainly types I and IV, and other dermal long-lived proteins such as fibronectin, are targets for glycation during intrinsic chronological ageing. Ultraviolet (UV) irradiation and smoking aggravate skin ageing, accelerate the formation of AGEs, and increase their deposition in tissues, including skin [101,102].

### 3.4. Inhibitors and Decoy Receptors for AGEs

In addition to RAGE, there are other receptors that bind AGEs but do not transduce cellular signals; thus, physiologically, they serve as decoy receptors or antagonists of AGE activity [98]. The receptors include macrophage scavenger receptors types I and II (SR-A, SR-BI, SR-BII, and CD36), stabilin-1, and stabilin-2 (Stab1 and Stab2) [99,100,101,102], oligosaccharyltransferase-4 (OST-48 or AGE-R1) [101], galactin-3 (AGE-R3) [102], protein kinase C substrate (AGE-R2) [103], and lectin-like oxidised LDL receptor-1 (LOX-1) [104,105]. They are mostly involved in modulating endocytosis and clearing AGEs. Their actions provide a potentially adaptive defence mechanism in the body to reduce the detrimental effects of elevated accumulation of glycation products, thus decreasing intracellular oxidative stress [9]. AGE-R1 is a subunit of N-oligosaccharyltransferase that belongs to a family of protein complexes responsible for the translocation of polypeptides across membranes [106]. Many chronic and age-related diseases reduce the expression of AGE-R1, such as diabetic nephropathy [107]. It is interesting to note that AGE-R1 is downregulated by high levels of AGEs [108] and in DM [109]. AGE-R1 overexpression in transgenic mice protected them against diabetic nephropathy [110] by preventing AGE accumulation [111] and increased AGE removal [112,113]. AGE-R2 is an 80–90 kD protein that contains a tyrosine-phosphorylated section responsible for intracellular signalling; it is phosphorylated upon exposure to AGEs. AGE-R2 signalling is thought to be important in the early stages of the AGE-induced response [98] by inducing cell activation to regulate AGE homeostasis [9,114]. Both AGE-R1 and AGE-R2 have been found in human atherosclerotic lesions with a distribution pattern similar to AGE accumulation in cells [114]. AGE ligands bind at the C-terminus of AGE-R3 with high affinity [102]. AGE–AGE-R3 binding increases the expression of AGE-R3, inducing a positive feedback loop by further increasing AGE–ligand binding and endocytosis by macrophages [37]. AGEs also bind to the class E scavenger receptor, LOX-1, and have been shown to increase LOX-1 expression in the vascular endothelium of diabetic rats [104,105]. AGE-R3-deficient diabetic mice suffer from glomerulopathy, proteinuria, and mesangial expansion [115].

There is also a circulating pool of RAGE, collectively known as sRAGE, and a minor product of alternative splicing of the gene encoding RAGE, known as endogenous secretory RAGE (esRAGE) [116]. These receptors are secreted and have been detected in human plasma [117]. sRAGE is generated by proteolytic cleavage of native membrane receptors mediated by matrix metalloproteinases (MMPs)—MMP-9 and disintegrin and metalloproteinase domain-containing protein 10 (ADAM10) [118,119,120]—or by alternative splicing to remove the transmembrane region [121]. Thus, sRAGE corresponds to the extracellular domain of RAGE (i.e., it lacks the cytosolic and transmembrane domains) [116,118,119,120]. This shedding of the receptor’s ectodomain is evoked by inflammatory stimuli, such as HMGB1 [117], lipopolysaccharide (LPS), and TNF-α [120]. RAGE activation stimulates MMP-9 expression and hence links these two forms of the receptor in the loop of autoinduction that may characterise states of low-grade chronic inflammation and unhealthy lifestyle [120,122].

The role of sRAGE is still debated because the available results are contradictory. In animal studies, administration of sRAGE prevented and slowed atherosclerosis [123], improved retinal neuronal dysfunction in diabetic mice [124], and accelerated wound healing in diabetic mice [125,126]. These results suggest that sRAGE acts by trapping, binding, and eliminating circulating AGEs. In human studies, sRAGE is considered a biomarker of adverse outcomes and disease risk due to the fact that elevated blood levels may reflect the amplification of pathological pro-inflammatory processes [127]. In several studies, the authors reported that in serum, the sRAGE levels are greatly increased and positively correlated with circulating inflammatory markers, such as TNF-α and MCP-1 in people with DM [128], diminished kidney function [129,130,131] and subclinical vascular disease [132]. Physical exercise has been reported to increase esRAGE levels in people at low/intermediate risk of CVD [133]. In contrast, multiple studies have shown decreased levels of sRAGE in patients with cardiometabolic and other chronic inflammatory conditions compared with healthy subjects [134,135,136]. To some extent, the explanation for this intriguing data may come from the above-mentioned interplay between AGEs, RAGE, MMPs, inflammatory molecules, and sRAGE and from the involvement of sRAGE in the oxidative stress response [137].

## 4. The Role of AGEs in Modulating the Immune System

AGEs affect immune cells that become dysfunctional as the human body ages. The formation of AGEs promotes the abnormal development of immune system cells and contributes to the violation of immune tolerance [138]. In the ageing organism, the pro- and anti-oxidant homeostasis is disturbed. With age, there is a significant increase in the production of ROS [139]. Key component of the adaptive immune system is CD4^+^ T (Th) helper cells. In adults, the differentiation and regulation of Th subsets are normal, thereby maintaining immune homeostasis, but in patients with DM, the balance is disturbed. Han et al. [140] have investigated that AGEs can induce the transition of naive CD4+ T cells to Th1 and Th17 cells, thereby promoting a pro-inflammatory environment. In addition, AGEs affect regulatory T (Treg) cells, weakening their suppressive effect. Stimulation of CD4^+^ T cells with AGE compounds resulted in increased expression of the RAGE in these cells, and knockout of RAGE by short hairpin RNA (shRNA) eliminated the effect of AGE on the differentiation of CD4^+^T cells and reduced the suppressive function of Treg cells [140].

CD14 (a co-receptor along with the Toll-like receptor [TLR4]) also performs an important role in the LPS-initiated signalling pathway for macrophage inflammatory responses. AGEs decrease the response to LPS in macrophages, which indicates AGE-related impairment of the immune response to microbial infection [141]. AGEs attenuate LPS uptake, impair C-X-C motif chemokine ligand 10 (CXCL10) production and reduce CD14 expression. Moreover, blocking RAGE activity restores LPS uptake, CD14 expression, and CXCL10 production [142].

Neutrophils can produce ROS and reactive nitrogen intermediates in the vascular compartment, a process mediated by NADPH oxidase and nitric oxide synthase (NOS), respectively. Hyperglycaemia in DM is responsible for stimulating circulating neutrophils, leading to excessive inflammation and tissue damage. AGEs may be responsible for the increased production of reactive oxygen and nitrogen species in neutrophils [143]. In addition, the accumulation of AGEs in the skin of diabetic rats may have an adverse effect on wound healing. Neutrophils are essential for the innate immune response after skin damage and act as a wound-healing initiator, leading to the accumulation and stimulation of macrophages and the removal of necrotic tissue. AGEs delay the migration and aggregation of neutrophils to the wound site, thus slowing down the healing process [144].

The immunomodulatory effect of AGEs has also been confirmed in a diabetic model using dendritic cells—antigen-presenting cells. The consequence of chronic hyperglycaemia is an increase in the number of dendritic cells and their impaired ability to stimulate primary immune responses. Price et al. [145] found that AGEs modulate the number, maturation, and function of peripheral blood dendritic cells and reduce their stimulating potential. This may explain the immunological consequences of the disease [145].

C-reactive protein (CRP) is a commonly used biomarker of ongoing inflammation in the body. One study conducted on people aged 50–73 years reported a significant correlation between higher CRP values and a diet rich in AGEs [146]. Similar results were obtained by Uribarri et al. [4]. Therefore, it can be argued that AGE-rich diets activate higher levels of CRP and may lead to inflammation, which could reflect an impairment of the innate immune response. This can be confirmed by the fact that people who consume more AGEs had a lower CD4/CD8 ratio and higher levels of B cells and natural killer (NK) cells. However, these differences were not statistically significant [146]. These studies are very promising, but more research and testing is needed.

## 5. AGEs in Ageing Skin

**Figure 2:**
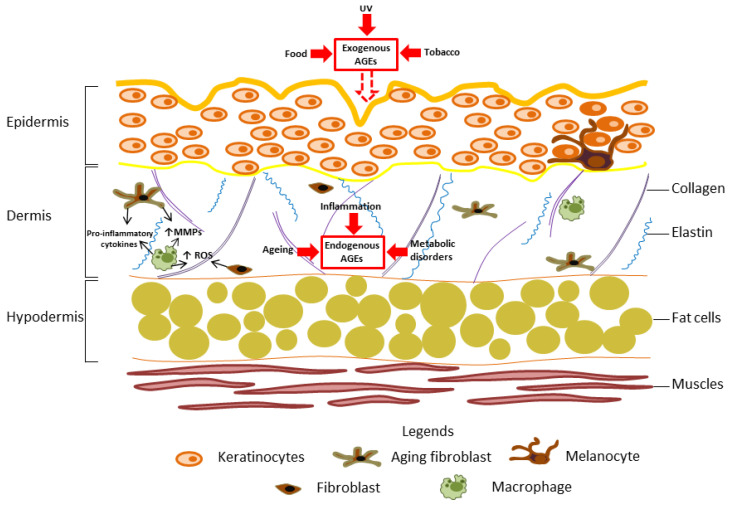
AGEs activity and impact on selected elements of ageing skin.

AGE accumulation in various tissues can be a marker of chronological ageing [147]. Characteristic findings of ageing skin, including decreased resistance to mechanical stress, impaired wound healing, and distorted dermal vasculature, can be partly attributable to the process of glycation and formation of glycation end products [148]. In ageing, the skin becomes dryer, thinner, and less elastic, and dark spots and wrinkles also appear [10,14]. Endogenous and exogenous AGEs perform an important role in this process. Endogenous AGEs are produced and accumulate in the skin during normal physiological metabolism with ageing or in diseases associated with inflammatory responses or chronic metabolic disorders. Exogenous AGEs are derived from diet, tobacco use, exposure to UV irradiation, and air pollution aid in their formation [149] (Figure 2).

AGEs bind to collagen and elastic fibres to reduce skin elasticity and to increase its rigidity. This phenomenon is mostly related to the changes in the synthesis of the ECM components and enzymes responsible for ECM turnover—that is, MMPs [10,149] (Figure 2). Chen et al. [150] showed that increased intake of dietary AGEs was positively correlated with the content of AGEs in the skin. UV irradiation and air pollution also increase AGE accumulation in the skin [39]. Sun-protected skin contains 10% less CML than sun-exposed skin. In addition, UV light influences CML and pentoside skin concentrations by inducing oxidative stress [151]. AGEs accumulate in photo-ageing skin, affect protein function in the dermis and promote skin ageing [152]. Tobacco is another factor that increases the concentration of AGEs in the skin. The tobacco metabolite nornicotine is involved in the synthesis of Amadori products, causing abnormal protein glycation, and the skin AGE fluorescence (SAF) value of smokers is significantly higher than that of non-smokers [153,154]. In another study, the skin level of AGEs in a group of 15 infants from smoking mothers was higher than in those from non-smoking mothers (86 infants) [155].

The levels of AGEs (CML, CEL, and pentosides) in skin collagen fibres increase linearly with age [91,149,156]. Verzijl et al. [91] showed that the long turnover time of collagen in the skin is the main reason for the accumulation of AGEs. Collagen is critical not only to the mechanical framework of the skin but also to several cellular processes; it is impaired by glycation in multiple ways [147]. Glycation modifies the biomechanical properties of collagen, resulting in increased stiffness and susceptibility to mechanical stimuli. Moreover, there is a change in the electrical charge of the protein, which interferes with its active sites, thus distorting the protein’s ability to properly interact with surrounding cells and matrix proteins and the ability to convert L-arginine to nitric oxide (NO), a critical cofactor in the cross-linking of collagen fibres, is impaired [157]. It results in skin dysfunction and decreases its regenerative potential [44]. Interestingly, Yoshinaga et al. [158] observed that CML-modified elastin is mostly found in sites of solar elastosis and is nearly absent in sun-protected skin. UV irradiation can mediate AGE accumulation in some capacity or, at the least, render cells more sensitive to external stimuli. In vitro studies have shown that CML, when bound to skin collagen, stimulates apoptosis in human fibroblasts through the activation of RAGE [159]. Due to the fact that RAGE is expressed specifically by keratinocytes, fibroblasts, and dendritic cells, RAGE activation contributes to changes in cellular activity and the production of cytokines and growth factors [10]. AGEs can also have an effect on intracellular processes. Glycation reduces the efficiency of proteasome enzymes, while accumulation of glycated vimentin within fibroblasts contributes to the decreased contractile activity of these cells in collagen gels [10]. In the skin, the intermediate filaments of fibroblasts (vimentin) and keratinocytes (cytokeratin 10) are susceptible to glycation modification [147]. Fibroblasts with glycated vimentin demonstrate a reduced contractile capacity, and this impairs their function [160]. In in vitro studies, high concentrations of AGEs cause human skin fibroblasts to show a higher rate of premature ageing and apoptosis. In addition, reduced synthesis of collagen and ECM proteins in cells and aged skin biopsies have been observed [147]. Furthermore, glycated collagen is highly resistant to degradation by MMPs [147]. An in vitro study using a three-dimensional model of reconstructed skin, with a dermal section in which the collagen had been modified by glycation, demonstrated a number of changes, including perturbations in MMP production, an increase in type IV collagen and laminin in the basement membrane and expansion of alpha 6 and beta 1 integrins in the suprabasal layers of the epidermis [161]. Interestingly, strict control of blood sugar for 4 months reduced the production of glycosylated collagen by 25%, and low-sugar food prepared by boiling could also reduce the production of AGEs [152].

Epigenetic factors, oxidative stress, UV irradiation and nutrition are important causes for the accumulation of chemically and structurally variable AGEs with different biological reactivities in the skin [10]. In one study, patients with severe psoriasis exhibited exacerbated production of endogenous AGEs, and the levels of AGEs in their skin were higher than those in the control group [162]. Interestingly, the fluorescent AGE concentration in the skin can be utilised to predict the occurrence of diseases, such as DM, kidney diseases, or CVD [163]. Gursinsky et al. [164] demonstrated that brief treatment of fibroblasts isolated from adult male Wistar rat hearts with fly ash triggered the immediate formation of intracellular ROS, and there was an increase in AGEs. In another in vitro study, Zhu et al. [165] demonstrated that AGEs in the epidermis could disrupt the migration and proliferation of keratinocytes, thereby resulting in a decreased ability of skin repair and impaired wound healing. In addition, the accumulation of AGEs could have a direct or indirect effect on skin pigmentation and its optical qualities [165]. 

## 6. AGEs and Vascular Dysfunction

Physiological changes to the vascular wall are dynamic and occur throughout life. Ageing is considered a prominent risk factor for CVD not just because of the cumulative effect of major risk factors over the passing years. Indeed, even in a perfect, healthy cardiovascular environment, ageing, by itself, progressively deteriorates cardiovascular homeostasis. Vascular ageing is characterised by a gradual change in the vascular structure and function, resulting in decreased arterial compliance and increased arterial stiffening [166]. Increased arterial stiffness is one of the earliest detectable changes in the structure and function of the vascular system and is considered a hallmark of vascular ageing. Age-related vascular changes accompany or even precede the development of AD pathology or hypertension [167,168]. Of note, arterial changes in young patients with hypertension mimic those in older normotensive individuals [168]. The molecular and cellular mechanisms underlying vascular alterations in ageing and hypertension are similar. They include aberrant signal transduction, oxidative stress, and activation of pro-inflammatory and pro-fibrotic transcription factors [168]. With ageing, there is a shift towards a pro-inflammatory vascular phenotype. There is significant upregulation of inflammatory cytokines, chemokines, adhesion molecules, and pro-inflammatory factors (MCP-1, transforming growth factor beta 1 (TGF-β1), MMP-2, AP-1, and NF-κB) in the vascular wall [169].

The endogenous formation of AGEs during physiological ageing is well described [170]. Due to their structure, blood vessels, especially arteries rich in collagen, elastin, and laminin, are the site of AGE accumulation and actions. The effect of the AGE–RAGE interaction on vascular complications has been best described in patients with T2DM. The chronic complications of T2DM are caused by structural and functional modifications of the vasculature. Structural modifications result from extracellular or intracellular proteins or polypeptides that are vulnerable to modification by AGEs [171]. Long-term complications seen in T2DM are mainly categorised as microvascular complications, such as diabetic retinopathy, nephropathy and peripheral neuropathy, and macrovascular complications, including CVD, cerebrovascular disease, and peripheral vascular disease [172,173]. Microvascular changes are discussed in Section 8 and Section 10. Macroangiopathy refers to damage in large- and medium-sized arteries, including the aorta, the coronary arteries, the carotid arteries, and arteries of the lower limbs. Medium- and small-sized arteries also experience medial calcification (medial calcinosis)—that is, a loss of elasticity in the media and internal elastic lamina [10]. Moreover, hyperglycaemia, insulin resistance, and excess fatty acids increase oxidative stress, disrupt protein kinase C (PKC) signalling, and increase AGEs, resulting in vascular inflammation, vasoconstriction, thrombosis, and atherogenesis [174]. Accelerated atherosclerosis in these patients is likely multifactorial. Intensive T2DM treatment produces a ≥10% risk reduction in major macro- and micro-vascular events [174]. AGEs induce the expression of pro-inflammatory mediators in various vascular cell types and are involved in a variety of microvascular and macrovascular complications [174]. Stirban et al. [175] demonstrated that in patients with T2DM, acute oral administration of a single AGE-modified protein class transiently but significantly impaired macrovascular function in concert with decreased NO bioavailability; these AGE-related changes were independent of heat treatment.

AGE accumulation has been strongly related to cardiac pathophysiology. The immune response and inflammation in the vasculature are key factors in the pathogenesis of CVD. Recently, the role of AGEs in diabetic cardiomyopathy has been characterised as triggering the production of NO and inducing ventricular remodelling [10]. AGEs participate in the progression of CVD by modifying extracellular and intracellular proteins, and signalling cascades via AGE–RAGE-activated pathways [176]. Elevated AGE levels have been associated with both systolic and diastolic dysfunction in patients with DM. It has been reported that AGEs accumulate in the myocardial interstitium between cardiomyocytes, affecting protein functions in the ECM of cardiac cells [170]. Furthermore, AGE levels in patients with DM have been shown to correlate with the degree of systolic dysfunction and indicators of diastolic dysfunction, such as delayed relaxation time and end-diastolic diameter [177]. AGE-lowering strategies (aminoguanidine, AGE breakers) have been associated with a reduction in heart stiffness and the development of cardiomyopathy in animal models [170]. The deleterious effects of AGEs on the heart have also been reported in non-diabetic models. Wall hypertrophy, increased heart volume, and a reduction in strain and the strain rate occurred in healthy Sprague Dawley rats that received daily intraperitoneal injections of AGE solutions for 6 weeks [178]. In another study, there was a significant reduction (40%) in left ventricular stiffness and an associated improvement in cardiac function in non-diabetic, non-obese, old dogs (about 10 years) treated for 1 month with an AGE cross-link breaker [179]. Several prospective studies have reported a positive association between plasma AGE levels and cardiovascular morbidity/mortality in patients with or without DM [170]. In addition, AGEs may perform a role in the development of coronary artery disease, both independently of and synergistically with T2DM [177].

Cardiac RAGE is predominantly expressed in the vascular endothelium and capillaries; it is also expressed in cardiomyocytes. The AGE–RAGE interaction may interfere with the calcium metabolism of cardiomyocytes and thus alter contractile function [180]. Post-translational modifications of sarcoplasmic reticulum proteins by AGEs may lead to alterations in calcium homeostasis and thus, abnormal cardiomyocyte relaxation and contractile dysfunction [181]. The induction of cardiac oxidative stress by AGEs has been reported as a trigger for ROS production, apoptosis, MAPK activation, nuclear O-linked β-N-acylation, and a reduction in endothelial NOS activity, which leads to enzyme uncoupling and ROS formation [170]. AGEs can also induce changes in the cytoskeleton of cardiomyocytes. In addition, the myocardium has many mitochondria and is particularly sensitive to the effects of AGEs on the respiratory chain and oxidative phosphorylation and can reduce the respiratory control ratio and activity of respiratory complexes in cardiomyocytes [182]. AGEs can also affect mitochondrial metabolism, in particular fission/fusion and degradation mechanisms by autophagy, which can lead to an energy imbalance responsible for cellular dysfunction [183].

Vascular ageing not only increases the risk of CVD (hypertension, coronary and peripheral artery disease, and stroke), but it also performs a major role in the aetiology of numerous common clinical disorders of advancing age, including chronic kidney disease (CKD), normal cognitive ageing and dementia, AD, metabolic disease, and reduced exercise capacity [184]. Stiffening of the arteries, formation of atherosclerotic plaques and endothelial dysfunctions are the main changes in the structure and function of the vascular system under the influence of AGEs [170]. Glycated extracellular proteins change the vascular stiffness and integrity of arteries [172]. In addition, AGEs can oxidise lipids. Glycated LDL leads to intracellular accumulation, foam cell formation and decreased NO production and LDL clearance [185]. Glycated high-density lipoprotein (HDL) also influences inflammation and reduces the removal of cholesterol and cholesterol transport. Together, glycated HDL and LDL promote atherosclerosis.

The AGE–RAGE interaction also influences vascular smooth muscle cells. Sakaguchi et al. [186] demonstrated that smooth muscle cell proliferation upon arterial injury is suppressed in RAGE-null mice compared with wild-type mice. Vascular endothelial cells form a monolayer inside the blood vessels and function as a barrier to regulate the permeation of blood components through the vessel wall. The permeability of the endothelial barrier is regulated by various cell–cell and gap junctions [173]. Disruption of this barrier can cause various pathologies. Furthermore, the AGE–RAGE axis in endothelial cells provokes the expression of genes such as *p22phox* and *gp91phox*, which are reduced forms of NADPH oxidase and cause endothelial cell dysfunction [172]. AGEs also perform a role in endothelial cell production of vascular endothelial growth factor (VEGF), which is involved in the development of atheroma [172]. The NF-κB–TNF–α–VEGF signalling cascade is activated by the AGE–RAGE axis and increases VEGF secretion, preventing the repair of endothelial lesions and inducing atherogenesis [187]. Therefore, AGEs increase vascular permeability. AGEs may degrade VE-cadherin by activating MMP-2 and MMP-9. In addition, ROS production induced by AGEs decreases the expression of VE-cadherin, β-catenin, and γ-catenin, and thus further enhances vascular permeability [188].

The soluble receptors for AGEs, sRAGE, and esRAGE, are believed to be the primary circulating decoy receptors that inhibit the interaction between membrane-bound (full-length) RAGE and its ligands, including AGEs [189]. sRAGE protects against AGE–RAGE-related pathologies, including vascular dysfunction and atherosclerosis, and obesity, by reducing AGE activity and signalling through the RAGE transmembrane effector [190,191]. In patients, a high number of risk factors, including hyperglycaemia, correlate with lower circulating sRAGE serum levels [192]. On the other hand, Dozio et al. [193] discovered a link between sRAGE, fibroblast growth factor 23 (FGF-23) and cardiovascular complications in patients with chronic diabetic nephropathy. sRAGE levels were higher than the normal range in both the DM and non-DM groups. However, because the ranges in the DM group were significantly higher than in the non-DM group, the author proposed that sRAGE may be a marker of cardiac remodelling [193].

## 7. AGEs in Ageing Ocular System

During physiological ageing, AGEs also gradually accumulate in the lens and retina, adversely affecting vision [194]. Recent findings show that N-(ε)-CML and AGEs are key modulators of the development of non-proliferative retinopathy in patients with T2DM and perform an important role in the development of microvascular complications of DM, including retinopathy and vascular occlusion and increased permeability of retinal endothelial cells. The AGE-RAGE interaction, by affecting inflammatory cells, such as macrophages and lymphocytes, and microvascular cells, such as endothelial cells or pericytes, have a significant impact on the progression of diabetic retinopathy [2]. These cells secrete inflammatory cytokines (IL-1, IL-6, IL-8, MCP-1, and TNF-α) by inducing NF-κB and upregulating adhesion molecules (VCAM and ICAM) [2]. Persistent inflammation negatively affects the retinal vasculature by altering the function of vascular cells. The consequence of the above changes is the thickening of the basement membrane and damage to the pericytes, which causes a vascular leak that destroys the retina [2,195]. In addition, macrophage inflammatory protein (MIP-1), IL-3, and IL-1 are thought to perform a role in angiogenesis, facilitating the transition from non-proliferative to proliferative diabetic retinopathy [2].

Glycation of the eye lens proteins is one of the mechanisms responsible for diabetic cataracts, which is the main cause of blindness [195]. AGEs cause irreversible changes in the structural proteins of the lens, which leads to the formation of high molecular weight aggregates that scatter light and impede vision [196]. AGE-modified proteins (glycation via pentosidine or CML) may become inactive. In this mechanism, the αA-crystallin protein undergoes conformational changes, which leads to the loss of chaperone functions. The consequence of these changes is widespread aggregation, protein insolubility, proteinopathy and cataracts. In turn, γB-crystallin, by aggregating, directly triggers the formation of cataracts [197].

## 8. AGEs in Brain Ageing

The brain is a high-energy-consuming organ that requires about 20% of the body’s basal oxygen to fulfil its functions. Thus, it is not surprising that disturbances in brain energy metabolism led to disease, ranging from subtle alterations in neuronal function to cell death and neurodegeneration. The brain seems to be particularly sensitive to the ageing process. Oxidative stress and oxidative protein damage can accelerate the formation of toxic protein oligomers that aggregate in the nucleus and cytoplasm of neurons. These changes are observed in normal ageing brains and age-related neurodegenerative disorders, such as AD and Parkinson’s disease (PD) [198]. As shown by Akhter et al. [199], in human and mouse brains, AGEs can be produced endogenously as a part of normal metabolism, and their accumulation is part of the normal ageing process; however, excessive accumulation of AGEs accelerates the ageing process and perturbs mitochondrial and synaptic function in nerve cells. Ahmed et al. [200] reported high concentrations of AGEs in cortical neurons and the cerebrospinal fluid of older adults. Moreover, it was demonstrated that the level of AGE adducts, including CML, CEL, and MG-H1, were significantly elevated in the cerebral cortex and hippocampus with advanced age in mice [199]. The highest levels occurred in 30-month-old mice. Accordingly, ageing mouse and human brains revealed decreased activities of mitochondrial respiratory chain complexes I, IV, and ATP levels and increased ROS [199].

Advancing age is not the only cause of the accumulation of AGEs. Chronic exposure to toxic agents, such as alcohol (ethanol), n-6 fatty acid–containing high-fat Western diets, and sugary soft drinks, also increase the accumulation of AGEs in the brain [201]. Oxidative stress can also be generated due to AGEs formation in the brain. This feed-forward process creates a vicious cycle that exacerbates oxidative damage with the subsequent initiation and progression of neurodegenerative diseases [201]. AGEs are major structural cross-linkers that can transform soluble neurofilament proteins into insoluble aggregates. Glycation is a spontaneous age-dependent post-translational modification that impacts the structure and function of several proteins. For example, glycation of α-synuclein with D-glucose results in misfolding and aggregation and might modulate the initial formation of aggregates, and glycation has been detected at the periphery of Lewy bodies in the brain of patients with PD at both the early and advanced stages. The presence of glycated forms of Aβ and tau in AD and the immunohistochemical localisation of AGEs suggest that protein glycation acts as a common detrimental posttranslational modification of major proteins associated with neurodegeneration [201].

It is known that the plasma levels of AGE differ in patients with AD or PD. Moreover, there are differences in the distribution of AGEs between the genders in patients with neurodegenerative diseases [201,202]. Activated human microglial cells produce and secrete AGE–albumin complexes, which increase RAGE expression in neurons. A recent study showed that RAGE is expressed in neural tissue, including neurons, microglia, astrocytes, and peripheral nerves [203]. In the cortex, hippocampus, cerebellum, and substantia nigra, RAGE promotes neuronal death, thereby contributing to neurodegenerative disorders. Immunohistochemical analysis of human primary neurons showed that the levels of p38K, p-p38K, SAPK/c-Jun N-terminal protein kinase (JNK), and p-SAPK/JNK were significantly increased, while the level of pERK1/2 was decreased after exposure to AGE-albumin [204]. These data indicate that AGE-albumin directly activates the MAPK pathway [205]. Additionally, the accumulation of AGEs and the AGE–RAGE interaction greatly induce reactive gliosis and the NF-κB pro-inflammatory pathway, leading to cellular stress, activated gliosis, and eventually neuronal degeneration [201].

Cerebral homeostasis is dependent on the integrity of the blood–brain barrier (BBB). This highly selective semipermeable physical and chemical barrier ensures the stable internal environment of the brain and prevents foreign object invasion. BBB dysfunction is involved in multiple human brain disorders, such as cerebral infarction, AD, and multiple sclerosis [206]. Researchers showed that exposure of human brain microvascular endothelial cells (HBMECs), one of the components of the BBB, to AGEs increased MMP-9 activation, leading to the degradation of neurotrophic tyrosine kinase receptor (TRKB) and brain-derived neurotrophic factor (BDNF) receptor. BDNF functions as a neuroprotectant through the activation of the TRKB [207].

**Figure 3:**
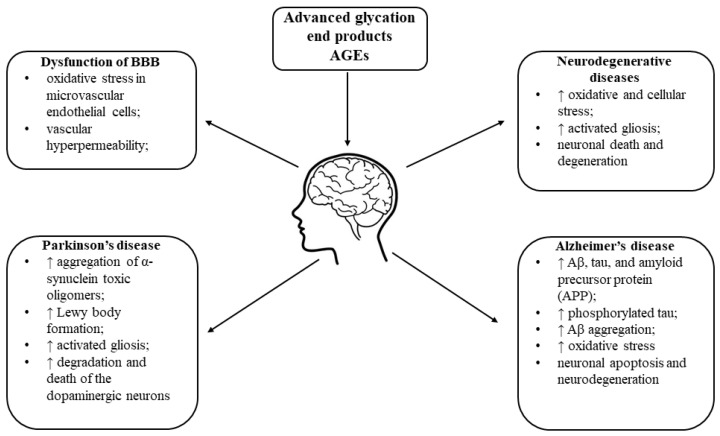
Advanced glycation end products AGEs and their causative functions in various ageing-related brain diseases. BBB; blood–brain barrier, ↑; increase, Aβ; amyloid β.

Moreover, it is known that AGEs induce oxidative stress in cerebral microvascular endothelial cells, thus contributing to vascular hyperpermeability. Dobi et al. [205] demonstrated that there is a relationship between cell barrier permeability, redox homeostasis, and mitochondrial respiration in brain microvascular endothelial cells exposed to AGEs. Exposure of brain microvessels to hyperglycaemic conditions or AGEs ex vivo resulted in significant abnormalities in the membranous distribution of tight junctions’ proteins. There was a significant increase in the number of extracellular vesicles (EVs) isolated from diabetic mice and higher expression of tight junctions’ proteins, occludin, and claudin-5 in EVs. Exposure of primary HBMECs to high glucose and AGEs led to significant augmentation of ICAM and VCAM expression, elevated leucocyte adhesion to and migration across HBMECs monolayers, and increased BBB permeability in vitro. Pericytes exposed to hyperglycaemia and AGEs displayed diminished expression of integrin α1, platelet- derived growth factor receptor- β (PDGF-R1β), and connexin-43 [208]. Figure 3 summarises how AGEs contribute to ageing-related brain diseases.

## 9. AGEs and Their Impact on Ageing-Related Changes in Pancreatic β-Cells

DM is a disease of advanced age associated with a negative impact on pancreatic cells. As mentioned previously, chronic hyperglycaemia is associated with a series of non-enzymatic glycation reactions with molecules, such as lipids, nucleic acids, and proteins. RAGE is also expressed in pancreatic β-cells [209,210]. AGEs contribute to the deterioration of pancreatic β-cell function by inhibiting the transcription of the insulin gene, degranulating β-cells, and ultimately reducing their total mass [211,212]. Studies in animal models have shown that reducing AGE intake significantly improves insulin sensitivity and extends the lifespan [213]. In patients with T2DM, AGE binding to RAGE can significantly accelerate the progression of the disease and cause complications with the vascular system and diabetic neuropathy and nephropathy [214,215,216].

Additional proof that AGEs may accelerate pancreatic β-cell dysfunction in patients with long-term hyperglycaemia comes from the study by Lim et al. [217]. The researchers noted increased ROS production in cells treated with AGEs compared to control cells treated with albumin. Moreover, RAGE was expressed in all β-cell lines and increased upon the addition of AGEs. In addition, TUNEL staining and flow cytometry with annexin V/PI antibodies showed that the number of apoptotic cells increased in AGE-co-incubated β-cell lines compared with control cells. Interestingly, the researchers also noted an increase in the cellular proliferation marker Ki67 in AGE-induced cells. It can be assumed that β-cell proliferation is a compensatory mechanism of AGE-related toxicity. In general, AGEs may cause deterioration of β-cell function due to their higher capacity to promote apoptosis than proliferation. Regulation of these processes is thought to be related to changes in the expression levels of RAD51 and RAD52 [217], which are involved in DNA repair. The NLRP3 (NLR Family Pyrin Domain Containing 3) inflammasome, which is an important component of the innate immune system, is involved in the pathogenesis of T2DM. It is assumed that the probable mechanism of AGE-induced islet damage is the activation of the NLRP3 inflammasome. The studies conducted in mice showed abnormal glucose tolerance, decreased insulin release, and ultrastructural pancreatic β-cell damage. Ablation of the NLRP3 inflammasome or treatment with the antioxidant N-acetylcysteine (NAC) markedly attenuated these effects [218]. Dietary AGEs have also been found to interfere with and downregulate sirtuin 1 (SIRT1) expression by causing acetylation and inactivation of peroxisome proliferator-activated receptor γ coactivator-1α (PGC1α), a master regulator of mitochondrial metabolism. AGEs also stimulate mitochondrial ROS production and JNK and NADPH oxidase activity to induce mitochondrial dysfunction and oxidative stress. In addition, increased oxidative stress and JNK/MAPK expression may contribute to pancreatic β-cell dysfunction and apoptosis, glucose-induced impairment of insulin secretion and subsequent insulin resistance, a key hallmark of T2DM [201,219]. AGEs have also been studied as possible biomarkers of gestational diabetes and complications at the end of pregnancy [220,221].

## 10. AGEs and Deterioration of Kidney Function with Ageing

Ageing is associated with significant changes in the structure and function of the kidneys, even in the absence of age-related comorbidities. The main pathological feature of the ageing kidney is nephrosclerosis, such as arteriosclerosis, glomerulosclerosis, tubular atrophy, and interstitial fibrosis. As a consequence of renal ageing, the number of nephrons decreases. A lower number of nephrons approximately parallels a decline in the glomerular filtration rate (GFR), and GFR declines with ageing. Recent studies have highlighted that decreased renal oxygen levels, mitochondrial dysfunction, and inflammation drive renal fibrosis as a mechanism involved in renal ageing [222].

The kidneys perform an important role in the clearance and metabolism of AGEs. They are particularly involved in the excretion of endogenous and dietary AGEs [170]. Renal clearance varies according to the molecular mass of AGEs and their protein-bound form—for example, the renal clearance of CML and MGO-lysine dimer have been reported as 4.2 ± 1.9 and 75 ± 22 mL/min, respectively, in healthy humans [223]. The kidneys also participate in AGE catabolism via the endolysosome system of proximal tubule cells, in which these products are reabsorbed after glomerular filtration [170]. Once filtered, AGEs bind to and/or enter proximal tubule cells, where their removal is dependent on lysosomal degradation and autophagy [224]. Fotheringham et al. [224] observed that in a human renal tubular epithelial cell line (HK-2 cells), exposure to AGEs significantly increased autophagosomes but markedly decreased autolysosomes. Moreover, oxidative stress evoked by AGEs performed an important role in the lysosomal dysfunction [224]. The kidneys are also a site for the accumulation of AGEs and AGE-associated damage [3]. CKD is associated with increased oxidative stress and the generation of AGEs, which in turn, contribute to the further decline of renal function [225]. AGEs are markedly elevated in the serum and tissues of patients with end-stage renal disease (ESRD) [226]. Hyperglycaemia is thought to increase the generation of endogenous AGEs, and the relationship between AGEs and renal disease has been studied extensively in patients with DM [227]. In 1991, Makita et al. [228] had observed that patients with DM and ESRD had twice the concentration of AGEs in tissues compared with patients with DM but without renal disease. Serum CML levels were 3–5-fold higher in patients with ESRD compared with healthy controls [3]. Later, Semba et al. [227] reported that elevated serum CML was associated with CKD. Moreover, the associations between CML levels and prevalent CKD in older adults at 3- and 6-year follow-ups were significant [227].

In humans, both CML and pentosidine accumulate in the expanded mesangial matrix and thicken glomerular capillary walls in early diabetic nephropathy and in nodular lesions and arterial walls in an advanced stage of disease [3]. In normal kidney homoeostasis, certain cell types, such as podocytes, endothelial cells, and mesangial cells express RAGE [229]. However, physiological RAGE expression on podocytes is relatively low [203]. The renal expression of RAGE is increased in diabetic nephropathy and in vitro in glomerular endothelial cells cultured with AGE–albumin [170]. Activation and upregulation of RAGE leads to multiple pathophysiological effects, including hypertrophy with cell cycle arrest and subsequent apoptosis, altered migration, and generation of pro-inflammatory cytokines. There is evidence that RAGE expression in renal endothelial cells performs an important role in renal vascular injury [203]. It has been observed that the AGE–RAGE interaction induces the expression of TGF-β1, a key mediator of renal fibrogenesis, in proximal tubule cells [3]. Moreover, this interaction induces apoptosis of podocytes and dysfunction of the glomerular filtration barrier. Consequently, the loss of podocytes leads to renal insufficiency and albuminuria [227]. Thieme et al. [230] performed in vitro studies in podocytes and showed that exposure to AGEs for 7 h decreased the expression of nephrin (*Nphs1*) but increased the expression of proteins involved in oxidative stress and fibrosis, such as thioredoxin-interacting protein (TXNIP), NADPH oxidase 4 (*Nox4*), and collagen type IV alpha 1 chain (*Col4a1*). Furthermore, epithelial-to-mesenchymal transition (EMT) markers, such as smooth muscle actin alpha 2 (*Acta2*), snail family transcriptional repressor 1 (*Snai1*), and the master regulator *Tgfb1* were also upregulated [230]. AGEs also decreased the glomerular content of a key epigenetic marker, namely, H3K27me3. Exposure to CML induced the transcription factor *Zeb2* through activation of the NF-κB signalling cascade in podocytes. *Zeb2* orchestrates EMT, during which cell–cell and cell–ECM interactions are weak and enable epithelial cells to become invasive. CML treatment induced both *NF-κB* and *Zeb2* promoter activity and suppressed E-cadherin promoter activity. Inhibition of *NF-κB* activity prevented CML-dependent induction of *Zeb2* and loss of E-cadherin. In vivo findings of elevated CML levels concurrent with increased expression of *Zeb2* in glomeruli and proteinuria in diabetic rats confirmed that CML mediated the effects under chronic DM conditions. These in vitro and in vivo studies suggest that the novel NF-κB–ZEB2 axis in podocytes performs a significant role in eliciting EMT and pathogenesis of diabetic nephropathy [231].

AGEs upregulate the synthesis of fibronectin, laminin, and type I and IV collagen in the kidney, promoting glomerular sclerosis, interstitial fibrosis, and hypertrophy [3,170]. In human proximal epithelial cells (HK-2), MGO-derived AGEs induced the expression of apoptotic markers, such as *Bax*, *p53* and kidney injury molecule-1, but downregulated *Bcl-2* and *cyclin D1* levels. Furthermore, MGO-derived AGEs induced mitochondrial dysfunction by promoting permeabilisation of the mitochondrial membrane and ATP synthesis [232]. Table 1 summarises the effects of the interaction of AGE with RAGE receptors in the renal system. 

In the kidney, AGEs can also bind to AGE-R1 and AGE-R3 [231]. AGE-R1 is expressed in glomerular cells, including podocytes and mesangial cells. It is postulated that in the podocytes AGE-R1 performs a role in the uptake and secretion of AGEs. The process whereby AGEs are cleared by AGE-R1 in the kidney is not well understood [236]. AGE-R1 is thought to be involved in the degradation of AGEs and excretion via the urine. Thus, there is a strong link between impaired podocyte function and albuminuria, increased urinary AGE excretion, and glomerulosclerosis [236]. In diabetic nephropathy, AGE-R1 expression is decreased, whereas upregulation of AGE-R1 augments the removal of AGEs. It is suggested that lowering the AGE burden, including facilitating renal AGE clearance, can be reno-protective in DM. An increase in podocyte AGE-R1 expression decreased renal AGE accumulation and increased urinary AGE excretion, but surprisingly, this was not associated with kidney protection. On the contrary, this change resulted in significant renal malfunction [236]. AGE-R3 is involved in facilitating the removal of AGEs and/or mediating the effects of AGE adducts in terms of cell activation and tissue injury induction [237]. Mice deficient in AGE-R3 developed glomerulopathy with a more pronounced increase in proteinuria, expression of ECM proteins and expansion of mesangial cells, all of which were associated with greater renal/glomerular AGE accumulation [237]. The association between a reduction in galectin-3 expression and greater susceptibility to AGE-induced renal disease, increased levels of AGE signalling and altered patterns of AGE-receptors activity suggest that AGE-R3 functions as an AGE receptor in vivo, thereby providing protection against AGE-dependent tissue injury [237].

## 11. AGEs and Cancer

Age is one of the main risk factors for cancer [23]. Moreover, it is generally accepted that chronic inflammation and oxidative stress, related to ageing, are intrinsically linked to cancer [23]. The potential contribution of AGEs to malignant cell transformation, development and progression of cancer also seems to be conclusive [23]. Compared to normal cells, cancer cells are characterised by increased glucose uptake and a high rate of glycolysis. As one of the consequence of an elevated glycolysis is the nonenzymatic glycation of proteins, this could lead to increased formation and accumulation of AGEs [23]. In 2005, van Heijst and co-workers [238], as a first, demonstrated the presence of AGEs on a series of human tumours of various origins. Using immunohistochemical staining, they assessed the expression of CML and argpyrimidine in squamous cell carcinomas of the larynx, adenocarcinomas of the breast, adenocarcinomas of the colon, and leiomyosarcomas. What is interesting different types of tumours accumulate different types of AGEs [238]. It is known that exposure of MDA-MB-231 breast cancer cell line to MGO increased proliferation, migration, and invasion of these cells. Moreover, MGO also increased the expression of the RAGE in breast cancer cell lines [239]. The strong association of RAGE expression with the malignant potential of gastric cancer, colon cancer, hepatocellular carcinoma, pancreatic cancer, prostate cancer, and oral squamous cell carcinoma was also discussed in the review by Malik et al. [240]. In vitro studies showed that silencing RAGE in human prostate cancer cells caused an inhibition of the proliferation and a decrease in the levels of prostate-specific antigen (PSA) [241]. In other reports, anti-RAGE antibodies inhibited tumour formation and lung metastasis of melanoma cell xenografts and subsequently improved survival in athymic mice [242]. All that evidence suggested that AGEs, via interaction with RAGE, could perform a role in various types of cancer and contribute to tumour development, migration, and metastasis. Another implication of AGEs in carcinogenesis is their ability to modify the ECM by establishing crosslinks that favour the invasion of cancer cells [241].

Interestingly, there are some studies which suggest substantial increase in cancer incidence in diabetic patients [243,244,245,246]. The worldwide prevalence of diabetes was estimated to rise from 171 million in 2000 to 366 million in 2030. About 26.9% of all people over 65 have diabetes, and 60% have cancer. Overall, 8–18% of cancer patients have diabetes [247]. It is well documented that longstanding type II diabetes is an established risk factor for cancer of the urinary tract, liver, biliary tract, pancreas, colon, endometrium, kidney, and breast [247]. It is also important to mention that treatment with metformin suppressed the expression of RAGE and cellular proliferation in breast cancer cell lines [241]. However, further mechanistic studies are warranted to establish biological pathways linking these two diseases and thereafter formulate efficient clinical preventive strategies.

## 12. AGE and Muscle Ageing

Skeletal muscles are one of the most abundant tissues in the human body. They are characterised by high dynamism and modulate many bodily functions. They are involved in the production of energy needed for movement, maintaining posture, breathing, generating heat, and maintaining homeostasis of metabolic processes [248]. Skeletal muscles significantly change their structure with age. Changes associated with muscle ageing begin in early adulthood and accelerate significantly later in life [249,250]. Elderly patients often experience musculoskeletal disorders that may be related to their AGE balance. These compounds accumulate in various neuromusculoskeletal tissues, where they adversely affect their biomechanical properties. As mentioned above, the AGE–RAGE interaction increases oxidative stress and activates the NF-κB pathway, which drives the secretion of pro-inflammatory cytokines and ROS production. As a consequence, muscle protein degradation and endothelial dysfunction in the skeletal muscle microcirculation system are accelerated [251,252,253]. Increased AGE-induced collagen cross-linking in the elderly has also been shown to stiffen the normally flexible ECM of skeletal muscle [254]. There is considerable evidence linking AGEs to sarcopenia [255]. This disease is associated with a decrease in muscle mass and a gradual impairment of muscle function. Thus, the life of elderly people becomes difficult: they are exposed to more frequent falls, weakness, hospitalisations, and reduced quality of life [256]. In a study conducted on the Japanese population, the authors found that AGEs can accelerate muscle wasting. The researchers enrolled 240 middle-aged and older adults (120 men and 120 women) in the study and measured their skin AGE accumulation by using the SAF index. In addition, they assessed other parameters related to sarcopenias, such as grip strength and the cross-sectional area (CSA) of the thigh muscles. They reported a correlation between a high SAF index and reduced grip strength, and a reduced thigh CSA. Interestingly, these observations concerned men but not women [257]. These results confirm earlier observations regarding the SAF index as a probable biomarker of sarcopenia risk [255,258,259]. In another study, the authors noted that elderly women with sarcopenia had significantly reduced weight, body mass index (BMI), bone density, appendicular lean mass, and percentage of lean body mass compared with the control group. In addition, among putative biochemical markers, there was a significant increase in plasma pentosidine. However, there were no significant differences in other biochemical parameters: pentosidine in urine, and homocysteine, 1,25(OA)2D and 25(OH)D. The study showed a correlation between plasma pentosidine levels and appendicular lean mass. There was no correlation between the SAF index and serum pentosidine, and these results suggest no relationship in the dynamics of the SAF index and serum pentosidine in sarcopenia in women [260]. Decreased voluntary muscle activation and increased antagonist co-activation may contribute to age-related muscle weakness. Arnold et al. [261] investigated the relationship between the profile of chronic low-grade inflammation and AGE (pentosidine) and selected parameters of muscle activation, which are likely to contribute to slowing down muscle efficiency. There was a negative relationship between MIP-1β (CCL4) and coactivation of the antagonist muscle during an isometric maximal isometric voluntary contraction (MVC), muscle activity of the antagonist muscle during the pre-movement time (PMT) and movement time (MT) during a fast dynamic contraction. In addition, elevated levels of pentosidine correlated with prolonged PMT. Therefore, it can be argued that pentosidine exerts both a mechanistic and inflammatory effect on slowing down upper limb movement with age [261]. In vitro and in vivo studies also provide evidence that AGEs are involved in skeletal muscle ageing. Egawa et al. [253] found that mice fed with a high-AGE diet for 16 weeks had less muscle mass, reduced muscle strength and less resistance to fatigue compared with mice fed a low-AGE diet. They found that the greater the increase in muscle AGE, the poorer the functional results [253]. Table 2 summarises the effects of AGEs on muscles.

## 13. Anti-AGEs Strategies

Increasingly broader knowledge about AGEs and the systemic threats they carry results in a significant increase in interest in the strategies preventing the formation of AGEs. These strategies focus on reducing their endogenous formation and accumulation. The main mechanisms that inhibit the formation of AGEs include the reduction in active dicarbonyl compounds, the inhibition of ROS formation, the protection of protein structure, and the degradation of AGEs [12,262]. For example, metformin lowers blood glucose levels and induces GLO1 activity, thereby reducing levels of MGO, an important precursor to AGEs. In turn, synthetic AGEs breakers, such as, for example, ALT-711 or Alt-TRC4186, can remove carbon-carbon bonds between carbonyl groups, thus removing cross-linked products. However, because these compounds cause adverse reactions, they cannot be used in the long run [12]. The search for natural substances in terms of new AGE inhibitors has been attracting more and more interest in recent years and indicates the possibility of using substances of natural origin. That compounds that inhibit the formation of AGEs are divided into six classes: polyphenols, polysaccharides, terpenoids, vitamins, alkaloids, and peptides [262,263,264]. These natural compounds are elegantly evaluated in the review by Anwar et al. [262]. It is important to look for new compounds that show good activity, efficiency, safety, and low incidence of adverse effects. Therefore, it seems that substances of natural origin may be a good source of new strategies limiting the formation of AGEs.

## 14. Lifestyle Interventions and AGEs

**Figure 4:**
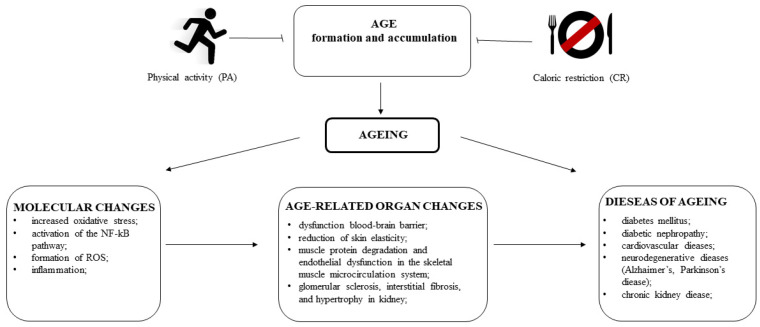
Lifestyle modifications, such as physical activity and caloric restriction, and their impact on human ageing are involved with AGE formation and accumulation. NF-kB; nuclear factor kappa-light-chain-enhancer of activated B cells, ROS; reactive oxygen species.

Ageing involves an overall change in a collection of physiological functions and an increased susceptibility to various diseases. As mentioned above, ageing itself is a condition that favours AGE formation and accumulation in many parts of the body, including the brain, blood vessel walls, skin, kidney, peripheral nerves, muscles, and pancreas. The build-up of these products results in significant changes in the metabolism, appearance, and biomechanical properties of these organs [10]. Therefore, inhibition of AGE formation might limit the progression of oxidative and inflammatory damage in tissues and improve the quality of life during ageing [265]. Recently, dietary modification and physical activity (PA) in people with and without age-related diseases have been reported to induce significant improvement in a series of functional and physiological parameters [266]. Figure 4 shows lifestyle interventions which help to decrease the AGE formation and accumulation, which consequently reduces cellular and organ changes accompanying aging.

### 14.1. The Relationship between PA and AGEs

The ageing population tends to include less PA in their daily routine, and they are more prone to chronic diseases and severe medical complications. Regular, moderate PA has an advantageous influence on health status and can reduce the risk and improve the prognosis of T2DM or CVD [267]. Regular PA is now considered an intervention to reduce AGE formation; it improves physical functioning and is essential for healthy ageing. Recent investigations on rodents have shown that PA reduces AGE concentration [268,269]. The levels of AGE precursors, such as MGO and 3-DG, in the renal cortex were normalised after 8 weeks of exercises in Zucker diabetic fatty (ZDF) rats [268]. Delbin et al. [269] investigated the effects of 8 weeks of aerobic exercise training on the vascular responses in isolated femoral and coronary artery rings from T1DM rats. AGE formation, measured by plasma CML levels, was significantly increased in the diabetic group compared with the control group. Exercise training reduced this effect by about 30% [269]. Moreover, RAGE protein expression in the coronary artery was reduced by approximately 46% in the trained group compared with the control group. Finally, Paramita et al. [270] showed that high-intensity interval training (HIIT) and moderate-intensity continuous training (MICT) reduced the expression of RAGE, NF-κB and TNFα in abdominal aorta tissue samples acquired from hyperglycaemic male Wistar rats.

The effects of PA have also been researched in human subjects. Researchers have shown that individuals who are regularly physically active have, on average, lower AGE levels than those that are not active [267]. Yoshikawa et al. [271] reported a significant reduction in blood AGE levels after exercise intervention in 47 middle-aged (35–70 years old) women without DM. The study group performed 60 min of walking per week for 12 weeks at 60% of their maximum heart rate (HRmax); the average number of steps per day significantly correlated with decreased CML levels. Another study showed that in patients with DM, total blood AGE levels were significantly reduced after resistance training [272]. Duda-Sobczak et al. [273] reported an association between self-reported PA and a marker of tissue accumulation of AGEs (skin autofluorescence) in patients with DM. They stated that higher PA is related to a lower accumulation of AGEs in adults with T1DM. In another study, regular exercise in patients with human immunodeficiency virus (HIV) reduced the serum AGE levels compared with inactive patients with HIV. Moreover, a combination of resistance and aerobic exercise three times per week for at least three months can lead to improvements in AGE outcomes for adults with HIV [274].

The mechanism by which AGE accumulation is reduced by regular exercise is not fully understood. Accumulated evidence has demonstrated that PA may affect circulating sRAGE levels [275,276,277]. Unfortunately, these studies provide conflicting results. For example, Kotani et al. [275] investigated the influence of 6 months of PA on circulating sRAGE in 30 community-dwelling asymptomatic Japanese volunteers (15 men and 15 women, mean age of 65 years). They showed a significant reduction in sRAGE levels during the intervention period. Farinha et al. [278] obtained similar results in insulin-dependent patients after high-intensity exercise training. Downregulation of sRAGE may be related to higher plasma total antioxidant capacity levels and enhanced antioxidant enzyme activity [278]. Exercise is thought to encourage more effective utilisation of reactive intermediates of the glycolytic and polyol pathways, thus reducing their availability to react with amino groups and to form AGEs [270]. On the other hand, two independent studies reported a significant increase in serum sRAGE levels [276,279]. More prospective studies are needed to better understand the impact of PA on AGE formation, tissue accumulation and receptor interaction.

### 14.2. CR as a Way to Reduce the Effects of AGEs

Reducing the number of calories inhibits premature ageing and significantly reduces the risk of age-related diseases [280]. CR from 25–60% of the energy demand significantly increases the lifespan and improves the quality of life of many animal species [280]. Moreover, CR is currently one of the most well-known and non-genetic interventions to delay the progression of ageing and the development of age-related chronic diseases. Of note, there are no side effects of this intervention. Over two decades ago, Teillet et al. [281] investigated the effect of chronic food restriction on AGE accumulation in lean female WAG/Rij rats as they aged from 10 to 30 months. They observed that 30% CR reduced the accumulation of AGEs and preserved the structure and function of the renal and cardiovascular systems. They observed similar effects in hepatocytes of lean ageing rats [282]. Sell et al. [283] observed that long term, 30% CR tended to decrease glycation of skin collagen in squirrels and rhesus monkeys. Moreover, RAGE expression was reduced in the gingiva of male F344BN male rats maintained on a CR diet compared to rats fed an ad libitum (AL) diet.

There are very limited data available regarding CR in humans. In 2004, Iwashige et al. [284] reported that in female Japanese patients with rheumatoid arthritis, after 54 days of CR, there had been a reduction in disease activity. That effect was accompanied by a reduction in urinary pentosidine levels. Moreover, in a population of people with overweight/obesity but otherwise healthy, weight loss induced by CR had a beneficial effect on serum AGE levels. After 2 months of the intervention, there had been a 7% reduction in the AGE levels. The decreased AGE concentration was significantly and positively correlated with that of triglycerides [285].

An important study modified AGE intake in 4-month-old C57BL/6 mice (n = 66). They were fed either a CR diet (40% calorie reduction) or a high-AGE CR diet in which AGEs were elevated by brief heat treatment. Detailed analysis showed that mice from the high-AGE CR group developed higher levels of 8-isoprostanes, AGEs, RAGE, and oxidative stress regulatory protein p66shc, coupled with low AGE-R1, GSH, and oxidised glutathione levels, decreased insulin resistance, marked myocardial and renal fibrosis, and a shortened lifespan. In contrast, control CR mice had lower oxidative stress markers, p66shc, RAGE and AGE levels, but high AGE-R1 levels, coupled with a longer lifespan [286]. These results support the hypothesis that oxidative stress can be reduced and the health span increased by consuming a diet where the AGE content is restricted. Another study reported the effect of 8 weeks of a low-AGE CR diet or a normal-AGE CR diet on glycaemic control, the lipid profile, inflammatory and oxidative stress biomarkers, and insulin resistance in non-smoking patients with overweight and metabolic syndrome (men and women, aged 18–70 years) [287]. The daily consumption of a low-AGE CR diet resulted in lower serum CML levels; reduced central obesity; and improved fasting blood glucose, insulin sensitivity, serum triglycerides levels, oxidative stress, and inflammatory markers, such as TNF-α.

Little is known about the molecular mechanisms by which CR influences AGE accumulation. One of the key molecules involved in this interaction is the deacetylase SIRT-1 [288], which links various signalling networks associated with ageing, such as NF-κB, 5’AMP- activated protein kinase (AMPK), mammalian target of rapamycin (mTOR), p53, PGC1α, and transcription factor -FoxOs [289]. Increased SIRT1 concentrations have been associated with better vascular homeostasis, metabolic profile, and protection against endothelial senescence. Rodents that received MGO supplementation had increased brain Aβ42 levels, AGE deposits, gliosis, and cognitive deficits, accompanied by suppressed SIRT1, nicotinamide phosphoribosyltransferase, AGE-R1, and peroxisome proliferator-activated receptor γ (PPARγ) levels [290]. Moreover, chronic consumption of high-AGE diets depleted defences, such as SIRT1 and PPARγ, predisposing to oxidative stress, inflammation, and chronic metabolic disease [291].

Roggerio et al. [288] examined the effects of CR (50%) and resveratrol on SIRT1 and esRAGE gene expression and serum levels in 48 healthy subjects with mild overweight (55–65 years old). The researchers noted a significant positive correlation between changes in SIRT1 and esRAGE gene expression after 30 days of CR. Moreover, CR increased serum levels of SIRT1. In the CR group, serum concentrations of esRAGE were correlated with esRAGE expression [288] in peripheral blood cells. In addition, the authors did not see any significant differences between CR and resveratrol administration.

Taken together, the available evidence has confirmed that CR is likely a way to lower exposure to dietary AGEs. The everyday diet is a continuous source of AGEs, and eating behaviour has a potential impact on the AGE concentration in the body. In general, AGEs are reduced when the overall calorie intake is decreased [277,287]. The reduction in energy intake lowers glucose in an organism and thus also reduces the levels of AGE substrates. In light of these observations, it has become important to study the link between CR and AGE accumulation given the major public health potential of dietary interventions, including CR, larger clinical trials are needed.

## 15. Conclusions

Ageing is a major risk factor for most chronic diseases and functional impairments. Age-related chronic diseases are becoming a major challenge for medicine and public health throughout the world. AGEs create crosslinks with many biomolecules, including proteins, and interact with plasma membrane local AGE receptors (RAGE), modulating intracellular signalling, expression of genes, and the production of free radicals and pro-inflammatory chemicals. Recent studies have provided evidence that AGEs are implicated in the development of chronic, degenerative diseases that occur in parallel to ageing. Due to the bioactivity, abundance and harmful potential of AGEs, it is critical to evaluate their role in health and disease, especially focusing on molecular ageing. Maintenance of cellular homeostasis and biomolecular stability and integrity is crucial for longevity and successful ageing. AGE accumulation dysregulates cellular physiology and signalling pathways and promotes remarkable alterations to normal tissue function. Moreover, AGEs, through the promotion of oxidative stress, activate several stress-induced transcription factors, including the MAPK, NF-κB, and STAT pathways, resulting in ROS production and aberrant secretion of proteins or growth factors. The AGE–RAGE interaction, triggering RAGE-linked mechanisms, and direct damage to the ECM are the two primary mechanisms by which AGEs alter tissue structures. The AGE–RAGE axis is disturbed in chronic, age-related diseases, such as DM, neurodegenerative diseases, CVD, and kidney disease.

To date, no single review article has discussed the role of caloric restriction and exercise in AGE reduction during age-related diseases and ageing. Previous reviews summarised the effect of potential pharmaceutical intervention strategies on the reduction in systemic AGEs burden [12]. Others focused on food-derived natural products, phytochemicals isolated from vegetables, legumes, fruits, or flavonoids that act as AGEs formation inhibitors, AGEs breakers, AGE–RAGE axis blockers, or glyoxalase stimulators [262,263]. In this review, we have attempted to provide an extensive analysis of the recent studies focusing on the different aspects of lifestyle interventions. By quoting original experiments made on animal models, we wanted to emphasise the fact that human intervention studies are required to investigate the beneficial effects of modest calorie restriction and physical activity in order to attain better, healthy ageing. We lack sufficient knowledge about AGEs; hence, future investigations are required to develop a greater understanding of the impact of AGEs on ageing processes.

The current evidence indicates that lifestyle modifications that promote a reduction in AGE accumulation are preferable and provide significant pro-health benefits. In our review, we shed more light on the application of physical activity and calorie restriction as a strategy to cope with the altered balance of AGEs accumulation and at the same time, minimise the AGEs-related conditions. Ageing is an inevitable, continuous process of life, but we may intervene to slow it down or improve the conditions of our ageing. The interventions (PA and CR) do not involve any extreme measures, but in the future, may greatly affect our well-being in advanced age.

## Figures and Tables

**Table 1:** The effects of the interaction of AGE with RAGE receptor in the renal system.

Type of Cell	Molecular Mechanisms	Effects
Endothelial cells	↑ VCAM1; ↑ ROS; ↑ VEGF R2; ↑ MMP2 and MMP9 are responsible for disrupting the tight junction [226]; affecting the cytoskeletal structure [227].	Aberrant angiogenesis [227]; ↑ permeability of glomerular filtration barrier [233,234].
Podocytes	↓ *Nphs1* expression [223]; ↑ expression of *Txnip*, *Nox4*, *Col4a1* [223]; upregulation of *Acta2*, *Snai1*, *Tgfb1* [220]; ↑ transcription factors *NF-kB* and *Zeb2* [224].	Induces apoptosis [203]; oxidative stress, fibrosis; EMT [230].
Mesangial cells	Induction of the expression of MCP-1 and VEGF; activation of NF-κB, MAPK, and PKC [228].	Hyperfiltration and microalbuminuria; promote proliferative inhibition, hypertrophy, and apoptosis [235].

↑ increase, ↓ decrease.

**Table 2:** The effect of AGE on muscular function.

Effects of AGEs on Muscles	Description of the Research Group	References
Decreased grip strength and thigh cross-sectional area in men	240 middle-aged and elderly people (120 women and 120 men)	[257]
Decreased grip strength and leg extension power	232 men were selected to measure grip strength and 138 men to measure leg extension strength	[255]
Decreased skeletal muscle index	132 participants (70 men, 62 women)	[258]
Decreased skeletal muscle index, grip strength, hip flexion strength	9203 participants	[259]
Decreased appendicular lean mass and percentage of lean body mass	47 women with sarcopenia, 23 healthy volunteers	[260]
Decreased muscle mass	mice with low and high AGEs diets	[253]

## Data Availability

Not applicable.
